# Fructose-containing sugars and metabolic risk: a systematic review and meta-analysis

**DOI:** 10.29219/fnr.v69.11062

**Published:** 2025-11-11

**Authors:** Prasetya Guntari, Astina Junaida, Palupi Eny, Namkieat Pichamon, Jiamjarasrangsi Wiroj, Sapwarobol Suwimol

**Affiliations:** 1 Department of Nutrition, STIKES Mitra Keluarga, Bekasi, Indonesia; 2 Noora Health Indonesia, Jakarta Selatan, Indonesia; 3 Department of Community Nutrition, Faculty of Human Ecology, IPB University, Bogor, Indonesia; 4 The Medical Food Research Unit, Department of Nutrition and Dietetics, Faculty of Allied Health Sciences, Chulalongkorn University, Bangkok, Thailand; 5 Department of Preventive and Social Medicine, Faculty of Medicine, Chulalongkorn University, Bangkok, Thailand

**Keywords:** fructose, glycaemic response, serum insulin, uric acid, meta-analysis

## Abstract

**Background:**

Fructose-containing sugars are widely consumed, yet their metabolic effects remain debated.

**Objective:**

This meta-analysis aimed to evaluate the impact of different fructose-containing sugars on glycaemic control, lipid profiles, and uric acid levels in adults.

**Methods:**

A total of 17 study codes from seven clinical trials were included, with intervention durations ranging from 7 h to 49 days. Interventions were classified as fructose, fructose-glucose mixtures (F/G), honey, or sucrose. Comparators varied and included unsweetened beverages, artificial sweeteners, and habitual diets. Meta-analyses using random-effects models assessed outcomes including fasting blood glucose (FBG), serum insulin, total cholesterol (TC), low-density lipoprotein cholesterol (LDL-c), very low-density lipoprotein cholesterol (VLDL-c), and uric acid. Effect sizes were reported as Hedges’ *g*.

**Results:**

Fructose-glucose mixtures intake significantly increased FBG (Hedges’ *g* = 0.474, *P* = 0.002) and serum insulin (Hedges’ *g* = 0.592, *P* < 0.001), while fructose, honey, and sucrose showed no significant effects. Monosaccharide intake modestly increased insulin (*P* = 0.006). Fructose and sucrose alone did not affect TC, but their combined intake resulted in a significant increase (Hedges’ *g* = 0.412, *P* = 0.009). No significant changes were observed in LDL-c, VLDL-c, or pooled metabolic outcomes. Fructose intake was strongly associated with increased uric acid (Hedges’ *g* = 1.628, *P* < 0.001), and pooled analysis of fructose, F/G, and honey also showed a significant increase (Hedges’ *g* = 0.550, *P* = 0.028).

**Conclusion:**

The short-term consumption of added sugars – fructose, sucrose, and F/G mixtures – had minimal effects on FBG, insulin, triglycerides (TG), non-esterified fatty acids (NEFAs), high-density lipoprotein cholesterol (HDL-c), and VLDL-c. However, significant increases in TC and LDL-c were observed, particularly with fructose and sucrose, indicating adverse effects on lipid metabolism. Some fructose interventions, especially those using high-fructose corn syrup, also showed marked increases in uric acid. While acute metabolic changes were limited, these findings suggest that regular intake of added sugars may elevate cardiometabolic risk. Long-term studies are warranted to clarify chronic effects and inform dietary guidelines.

## Popular scientific summary

- Fructose shows no effect on blood glucose and serum insulin while sucrose consumption posted a significant effect.
- Fructose bypasses the phosphofructokinase as the major rate-limiting step of glycolysis. It therefore can be used as the substrate for glycerol synthesis or fatty acids through de novo lipogenesis (DNL).
- Fructose consumption posted a positive strong correlation with serum triglycerides.
- High fructose corn syrup had a remarkable effect on serum uric acid.

The ‘pandemic obesity’ phenomenon has been associated with increased consumption of foods and beverages containing added sugars, particularly fructose. Between 1977 and 2001, daily energy intake among Americans aged 2 years and older rose by an estimated 150–300 kcal (1–4). Notably, up to 50% of this increased caloric intake has been attributed to the consumption of sugar-sweetened beverages (SSB), with a significant contribution from fructose-containing drinks (3). An average daily fructose intake in the United States (US) and several European countries has been sustained at 50–60 g for over three decades (5, 6).

Concurrently, global data from 185 countries reveal an increasing trend in SSB consumption, with an average weekly increment of 0.37 servings. This pattern is particularly marked in sub-Saharan Africa (7). Observational studies have validated these concerns, demonstrating that excessive fructose intake contributes to the global prevalence of obesity, diabetes mellitus (DM), and associated cardiometabolic risk factors. Furthermore, the unique metabolic pathways of fructose may induce significant metabolic derangements, including dyslipidemia, hyperuricemia, and hepatic steatosis in humans (8–12). In addition, the research done by Erkkila et al. (13) involved a study on the effects of moderate increases in dietary sucrose on serum lipids. The study found that higher intakes of added sugars, particularly from SSBs and foods high in fructose, were positively associated with several indicators of the metabolic syndrome, including increased waist circumference, elevated triglycerides (TG), and reduced high-density lipoprotein cholesterol (HDL-c). These findings support the hypothesis that excessive intake of fructose-containing sugars may contribute to the development and progression of metabolic abnormalities (13).

Fructose is metabolised differently from glucose, primarily in the liver via the enzyme fructokinase. Unlike glucose, which is processed throughout various tissues, fructose metabolism occurs predominantly in the liver. Excessive fructose intake activates lipogenic enzymes, such as fatty acid synthase and acetyl-CoA carboxylase, promoting de novo lipogenesis (DNL). This process leads to the accumulation of lipid droplets in the liver (14). Consequently, fatty acid oxidation is inhibited, exacerbating intrahepatic lipid accumulation, which in turn promotes the production of very low-density lipoprotein 1 (VLDL1) and elevates postprandial TG levels (15).

Furthermore, the accumulation of lipids in the liver contributes to the development of hepatic insulin resistance. This occurs through the serine phosphorylation of the insulin receptor and insulin receptor substrate 1 (IRS-1), impairing the insulin signalling pathway. Hepatic insulin resistance not only disrupts glucose metabolism but also further exacerbates DNL and upregulates apo-B synthesis (15). This feedback loop increases very low-density lipoprotein cholesterol VLDL-c secretion, and leads to hypertriglyceridemia as well as metabolic syndrome.

However, the precise effects of fructose on lipogenesis and the development of metabolic syndrome remain incompletely elucidated, particularly in the context of clinical studies. Existing meta-analyses examining these relationships in randomized controlled trials (RCTs) are limited. Therefore, this meta-analysis and systematic review aimed to quantitatively evaluate the effects of dietary fructose consumption on metabolic biomarkers associated with lipogenesis and metabolic syndrome in RCTs.

## Methods

A systematic review and meta-analysis was conducted in accordance with the Cochrane Handbook for Systematic Reviews of Interventions (version 6.3) (16) and reported in compliance with the Preferred Reporting Items for Systematic Reviews and Meta-Analyses (PRISMA) guidelines.

The study was registered with PROSPERO (http://www.crd.york.ac.uk/PROSPERO/), registration number CRD42022293967, on 15 May 2023. Electronic searches were performed to identify relevant research articles from MEDLINE (PubMed), ScienceDirect, and the Cochrane Central Register of Controlled Trials databases, covering the period from October 2017 to April 2023. The search strategy employed the keywords ‘fructose’ AND (‘lipogenesis’ OR ‘metabolic syndrome’), excluding animal studies. Limiting the search to English-language publications yielded 175 potential references in the initial screening phase.

The literature selection process employed the following inclusion criteria:

1. Full-text articles published in English.
2. Articles published in peer-reviewed journals.
3. Studies providing a direct comparison between fructose and other sugar-containing foods or beverages (glucose, sucrose, fructose-glucose mixtures [F/G], or honey).
4. Randomized controlled trials or crossover clinical trials conducted in healthy adult or older adult populations.

Two independent groups of reviewers screened the titles and abstracts of retrieved studies based on predefined inclusion criteria. Full-text articles of potentially eligible studies were subsequently reviewed independently by the same reviewers to determine final eligibility for inclusion in the systematic review.

**Fig. 1:**
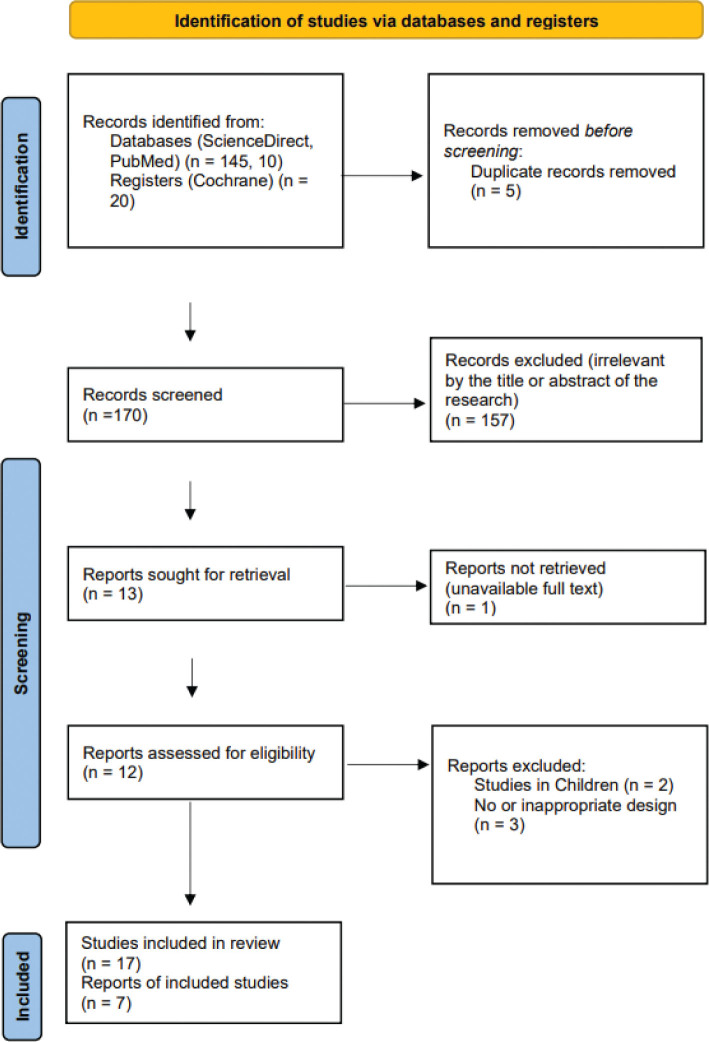
PRISMA flow diagram.

The initial title screening identified 175 potentially relevant articles. After removing 5 duplicate records, 157 articles were excluded based on irrelevance determined from titles and abstracts. Of the 13 articles retained for full-text review, 6 were further excluded due to the following reasons: inclusion of paediatric populations (*n* = 2), unavailability of full text (*n* = 1), and use of intervention regimens not aligned with the study criteria (*n* = 3). Consequently, 7 articles met the inclusion criteria and were included into the final data extraction and statistical analysis (Fig. 1).

### Studies coding

A total of 17 studies were identified within the 7 selected articles. Data were coded based on the number of treatment arms. Study variations were characterised by factors including sugar type or composition, participant gender, intervention duration, nutritional status, and intervention dosage. Initial study details, such as author(s), publication year, study design and location, and participant nutritional status, were recorded and summarised in Tables 1 and 2. Mean values and standard deviations for each measured parameter (fasting blood glucose [FBG], serum insulin, total cholesterol [TC], HDL-c, low-density lipoprotein cholesterol [LDL-c], TG, non-esterified fatty acids (NEFAs), and uric acid) were extracted for tabulation. Prior to statistical analysis, all data were converted to standardised units of measurement.

**Table 1:** List of comparison studies used in meta-analysis

Study code	Author	Study design	Duration of study	Place of research	Nutritional status
1	Geidl-Flueck et al. (14)	RCTs	49 days	Switzerland	Normal
2	Geidl-Flueck et al. (14)	RCTs	49 days	Switzerland	Normal
3	Debray et al. (20)	Crossover controlled trial	7 days	Switzerland	Normal
4	Debray et al. (20)	Crossover controlled trial	7 days	Switzerland	Normal
5	Hieronimus et al. (21)	Parallel arm trial	14 days	USA	Normal
6	Hieronimus et al. (21)	Parallel arm trial	14 days	USA	Normal
7	Hieronimus et al. (21)	Parallel arm trial	14 days	USA	Normal
8	Hieronimus et al. (21)	Parallel arm trial	14 days	USA	Normal
9	Varsamis et al. (22)	Randomized cross-over trial	7 h	Australia	Overweight/Obese
10	Varsamis et al. (22)	Randomized cross-over trial	7 h	Australia	Overweight/Obese
11	Damiot et al. (23)	RCTs	10 days	France	Normal
12	Damiot et al. (23)	RCTs	10 days	France	Normal
13	Low et al. (24)	Randomized cross-over study	2 days	UK	Obese
14	Low et al. (24)	Randomized cross-over study	2 days	UK	Obese
15	Low et al. (24)	Randomized cross-over study	2 days	UK	Obese
16	Despland et al. (25)	A randomized, open-label, cross-over CT	8 days	Switzerland	Normal
17	Despland et al. (25)	A randomized, open-label, cross-over CT	8 days	Switzerland	Normal

**Table 2:** Intervention detail of studies used in meta-analysis

Study code	Type of sugar	Type of intervention given	Type of control given	Duration of study	Dose (g) of fructose		Sex	Age (years)	Number of subjects	
					Control	Intervention			Control	Intervention
1	Fruc	Fructose	Unsweetened beverage	49 days	0	80	Male	18–30	24	23
2	Suc	Sucrose	Unsweetened beverage	49 days	0	80	Male	18–30	24	23
3	Fruc	High fructose diet	Low fructose diet	7 days	10	100.13	Male and female	40.68	6	6
4	Fruc	High fructose diet	Low fructose diet	7 days	10	101.89	Male and female	40.93	6	6
5	Fruc	Fructose-25%	Aspartame	14 days	0	109	Male and female	26.10	23	28
6	Fruc	HFCS-25%	Aspartame	14 days	0	133	Male and female	26.10	23	28
7	Fruc	Fructose-17.5%	Aspartame	14 days	0	74	Male and female	25.49	23	22
8	Fruc	HFCS-17.5%	Aspartame	14 days	0	91	Male and female	24.59	23	16
9	F/G	SSB	Water consumption	7 h	0	42.11	Male and female	19–30	28	28
10	F/G	SSB	Water consumption	7 h	0	42.11	Male and female	19–30	28	28
11	F/G	Fructose mixed with glucose	Free-living diet	10 days	40	232.5	Male	N/A	10	10
12	F/G	Fructose mixed with glucose	Free-living diet	10 days	40	232.5	Male	N/A	10	10
13	Fruc	High fructose drink	Low fructose drink	2 days	20	60	Male and female	44.7	16	16
14	Fruc	High fructose drink	Low fructose drink	2 days	20	60	Male	42.8	8	8
15	Fruc	High fructose drink	Low fructose drink	2 days	20	60	Female	46.6	8	8
16	Honey	Honey (high in fructose)	Low sugar	8 days	9.5	95	Male	NA	8	8
17	F/G	High fructose with glucose	Low sugar	8 days	9.5	47.5	Male	NA	8	8

### Statistical analysis

Data analysis was performed using Hedges’ *g* to quantify the effect size and assess the difference between intervention/treatment and control groups. Hedges’ *g* allows for the calculation of effect size independent of variations in sample size, measurement units, and statistical test results. Furthermore, this method is particularly suitable for estimating the effect of paired treatments, as demonstrated in prior research (17, 18). The intervention effect was determined by comparing the treatment group (E) to the control group (C). A positive effect size indicates a greater observed parameter value in the treatment group, whereas a negative effect size indicates a lower value. The effect size (d) was calculated using the following formula:


$$d\;(effect\;size)=\frac{{\underline{X}}^{E}-{\underline{X}}^{C}}{s}J$$ (1)

where ${\underline{X}}^{E}$ is the mean value from experimental group, ${\underline{X}}^{C}$ is the mean value of control group. *J* is the correction factor for small sample size, that is:


$$J=1-\frac{3}{4(N^{C}+N^{E}-2)-1}$$ (2)

and *S* is the pooled standard deviation, defined as:


$$S=\sqrt{\frac{(N^{E}-1)(s^{E}{)}^{2}+(N^{C}-1)(s^{C}{)}^{2}}{(N^{C}+N^{E}-2)-1}}$$ (3)

where *N^E^* is the sample size of the experimental group, *N^E^* is the sample size of the control group, *S^E^* is the standard deviation of the experimental group, and *S^C^* is the standard deviation of the control group. And the **variance of Hedges’ *g*** (*v_d_*) is described as:


$$V_{d}(Varians)=\frac{N^{C}+N^{E}}{N^{C}N^{E}}+\frac{d^{2}}{\left(2(N^{C}+N^{E})\right)}$$ (4)

$$d_{++}(cummulative\;effect\;size)=\frac{\left(\sum_{i=1}^{n}w_{i}d_{i}\right)}{\left(\sum_{i=1}^{n}w_{i}\right)}$$ (5)

where *w_i_* is the inverse of the sampling variance: $w_{i}=\frac{1}{V_{d}}$.

The precision of the effect size is described by using **95% of CI** (confidence interval), that is:


$$d\pm (1.96x_{d}^{S}).$$

The formulas employed in the aforementioned equations are derived from the work of Rosenberg et al. (19) and Sánchez-Meca and Marín-Martínez (18). Statistical significance of the effect size was determined by the absence of a null effect size within the CI (18). To assess potential publication bias from unpublished or unanalysed studies, a fail-safe number was calculated. A fail-safe number exceeding 5N+10, as determined by Rosenthal’s method (19), indicates a robust meta-analysis model.

The magnitude of the effect size was interpreted using Cohen’s benchmarks, with small, medium, and large effects defined as 0.2, 0.5, and 0.8, respectively (19). Cumulative effect sizes were calculated for distinct variable clusters, including season, processing unit, and survey type. All effect size calculations were performed using Comprehensive Meta-Analysis software, and were restricted to studies reporting sample size, mean, and standard deviation.

## Results

This meta-analysis included data from 17 study codes, derived from seven clinical trials. The duration of these studies ranged from acute (7 h) to medium-term (up to 49 days). The majority of studies involved normoweight participants; only five study codes included overweight or obese individuals (Table 1). Intervention details for each study are presented in Table 2. Based on the administered intervention, sugar types were classified as fructose, F/G, or honey. Control groups varied across included studies, encompassing unsweetened beverages, aspartame, low-sugar or low-fructose diets, and habitual dietary patterns. Age and gender were not controlled for in this analysis, as the included studies comprised both adult males and females.

### Effects of fructose-glucose mixtures, fructose, honey, and sucrose on fasting blood glucose

**Fig. 2:**
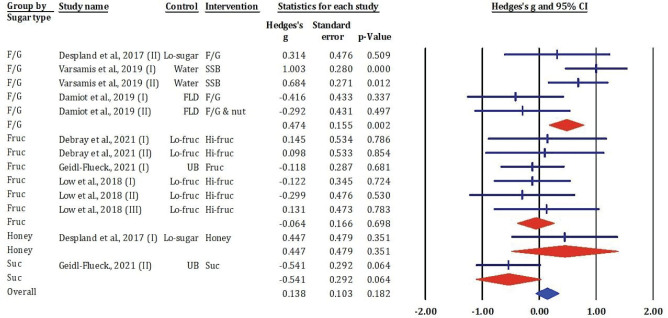
Effects of F/G, fructose, honey, and sucrose on FBG: Meta-analysis results (*n* = 13).

Figure 2 illustrated a forest plot summarising the standardised mean differences (Hedges’ *g*) and 95% CIs for the effects of various sugar types – F/G, fructose Fruc, honey, and sucrose – on FBG levels, based on data from 16 comparisons.

Within the F/G subgroup, two studies (22 [I and II]) reported significant increases in FBG following SSB consumption (Hedges’ *g* = 1.003 and 1.084; both *P* < 0.05), indicating a strong glycaemic impact. In contrast, other comparisons in the F/G group reported non-significant effects, including both negative and positive changes in FBG (e.g. 23, 25).

In the fructose subgroup, all comparisons (14, 20, 24) showed small and statistically non-significant changes in FBG (Hedges’ *g* ranging from −0.209 to 0.145; all *P* > 0.05). Similarly, studies involving honey (25) and sucrose (14) indicated small to moderate changes in FBG, with none reaching statistical significance.

The overall pooled estimate revealed a small, non-significant increase in FBG across all sugar types (Hedges’ *g* = 0.138, 95% CI: −0.065 to 0.341, *P* = 0.182). These findings suggest that while certain F/G interventions, particularly SSBs, may significantly raise FBG, most sugar types studied do not produce a consistent or statistically significant effect on fasting glucose concentrations.

### Effects of fructose-glucose mixtures, fructose, honey, and sucrose on serum insulin

**Fig. 3:**
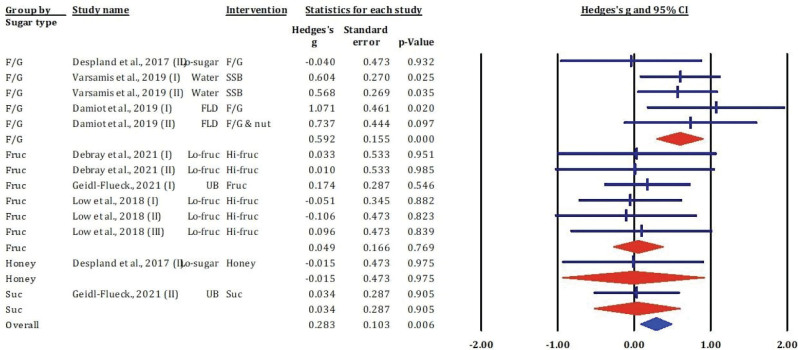
Effects of F/G, fructose, honey, and sucrose on serum insulin: meta-analysis results (*n* = 13).

Figure 3 presents a forest plot summarising the effects of various dietary sugar types – including F/G, fructose, honey, and sucrose – on serum insulin concentrations. The analysis includes 16 comparisons, with results expressed as standardised mean differences (Hedges’ *g*) and 95% CIs.

In the F/G subgroup, Varsamis et al. (22, I and II) showed significant increases in serum insulin after SSB consumption (Hedges’ *g* = 1.003 and 1.084, both *P* < 0.05), indicating a robust insulinotropic effect. In contrast, other studies in the same subgroup (23, 25) reported non-significant changes, with both negative and positive effect sizes, suggesting heterogeneity in response depending on formulation or co-interventions (e.g. with nuts or fibre).

For the fructose subgroup, all included studies (14, 20, 24) showed small and non-significant effects on serum insulin (Hedges’ *g* from −0.209 to 0.145; *P* > 0.05), indicating no clear stimulatory or suppressive action of fructose on insulin levels in these contexts.

Honey intake (25) produced a small to moderate, but statistically non-significant, increase in insulin (Hedges’ *g* = 0.447; *P* = 0.351). For sucrose, Geidl-Flueck et al. (14) observed a moderate reduction in insulin (Hedges’ *g* = −0.541), approaching significance (*P* = 0.064), though the limited data warrant cautious interpretation.

The pooled effect across all studies showed a small, non-significant increase in serum insulin levels (Hedges’ *g* = 0.138, 95% CI: −0.063 to 0.339, *P* = 0.182), suggesting that, overall, sugar type did not significantly influence insulin concentrations under the conditions examined.

### Effects of fructose-glucose mixtures, fructose, honey, and sucrose on total cholesterol

**Fig. 4:**
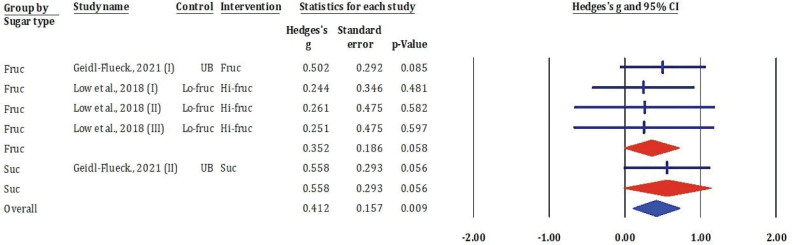
Effects of F/G, fructose, honey, and sucrose on TC: meta-analysis results (*n* = 5).

Figure 4 showed the results from meta-analysis synthesised data from six RCTs investigating the effects of different sugars – fructose, sucrose, and F/G – on TC levels. Individual study effect sizes were calculated using Hedges’ *g*, with 95% CIs represented in the forest plot.

Among the five fructose-related interventions, three were derived from Low et al. (24) and two from Geidl-Flueck et al. (14). The pooled effect for fructose consumption showed a small-to-moderate increase in TC (Hedges’ *g* = 0.352, standard error [SE] = 0.186, *P* = 0.058), although statistical significance was marginal. In contrast, sucrose interventions showed a stronger and more consistent effect across two comparisons, both reporting Hedges’ *g* values above 0.55 and reaching borderline significance (*P* = 0.056).

The overall pooled estimate from all included studies demonstrated a significant increase in TC following sugar intervention compared to control (Hedges’ *g* = 0.412, SE = 0.157, *P* = 0.009), suggesting that both fructose and sucrose contribute to elevated TC levels. The 95% CI for the overall effect did not cross zero, reinforcing the robustness of the association.

### Effects of fructose-glucose mixtures, fructose, honey, and sucrose on high-density lipoprotein cholesterol

**Fig. 5:**
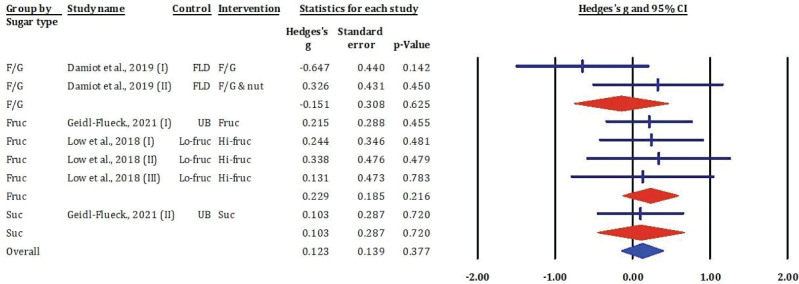
Effects of F/G, fructose, honey, and sucrose on HDL-c: meta-analysis results (*n* = 7).

Figure 5 presented the results of meta-analysis included seven comparisons from RCTs assessing the impact of different dietary sugars on HDL-c concentrations. The overall pooled effect across all studies was not statistically significant (Hedges’s *g* = 0.123, SE = 0.139, *P* = 0.377), indicating that sugar consumption had no appreciable effect on HDL-c levels.

Subgroup analyses revealed variable results. Interventions with F/G yielded mixed outcomes, with one study reporting a moderate decrease (Hedges’s *g* = -0.647, *P* = 0.142) and another reporting a small increase (Hedges’s *g* = 0.326, *P* = 0.450), though neither reached statistical significance. Fructose-only interventions consistently showed small, positive, but non-significant effects on HDL-c (Hedges’s *g* ranging from 0.131 to 0.335). Similarly, sucrose interventions demonstrated negligible effects (both Hedges’s *g* = 0.103, *P* = 0.720).

Overall, the evidence does not support a significant effect of dietary fructose, sucrose, or F/G mixtures on HDL-c concentrations in adults. These findings suggest that while added sugars influence other lipid markers, their impact on HDL-c is minimal and inconsistent across studies.

### Effects of fructose-glucose mixtures, fructose, honey, and sucrose on low-density lipoprotein cholesterol

**Fig. 6:**
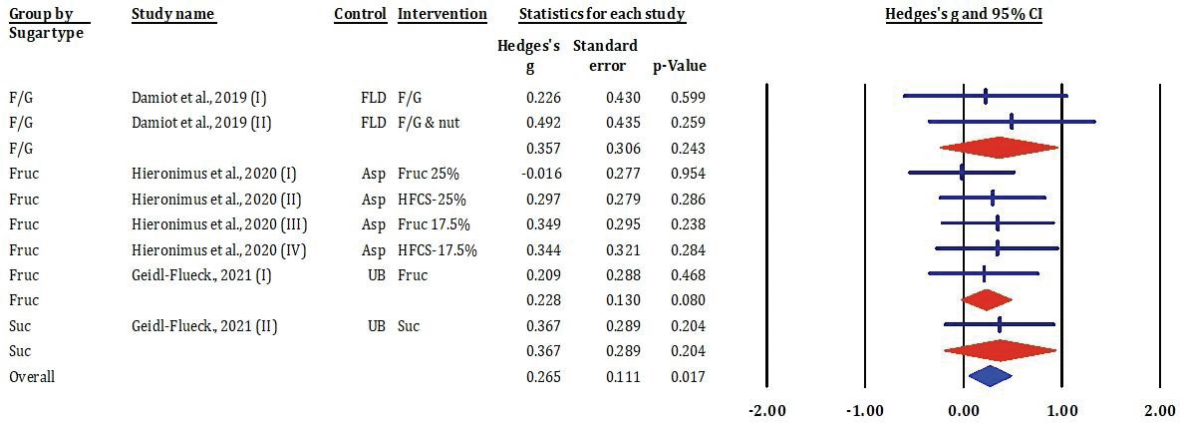
Effects of F/G, fructose, honey, and sucrose on LDL-c: meta-analysis results (*n* = 8).

Figure 6 showed the results of meta-analysis included eight comparisons examining the effects of various dietary sugars on LDL-c. The overall pooled analysis demonstrated a small but statistically significant increase in LDL-c following sugar consumption (Hedges’s *g* = 0.265, SE = 0.111, *P* = 0.017), suggesting that added sugars contribute modestly to LDL-c elevation.

Subgroup analysis revealed heterogeneous but generally positive effects across sugar types. Fructose-glucose mixtures produced variable outcomes, with Hedges’s *g* values ranging from 0.226 to 0.492; however, none reached statistical significance (*P* > 0.05). Fructose-related interventions, including high-fructose corn syrup (HFCS) at varying concentrations, showed consistent trends towards increased LDL-c, with effect sizes between 0.209 and 0.349, though none were individually significant (*P* > 0.2). Sucrose interventions, both from a single study (14), yielded a pooled Hedges’s *g* of 0.367 with borderline significance (*P* = 0.204).

Despite the variability in individual study outcomes, the significant overall effect suggests that regular intake of added sugars – particularly fructose and sucrose – may contribute to atherogenic lipid changes, notably increased LDL-c, which is a key risk factor for cardiovascular disease.

### Effects of fructose and glucose mixture, fructose, honey, and sucrose on very low-density lipoprotein cholesterol

**Fig. 7:**
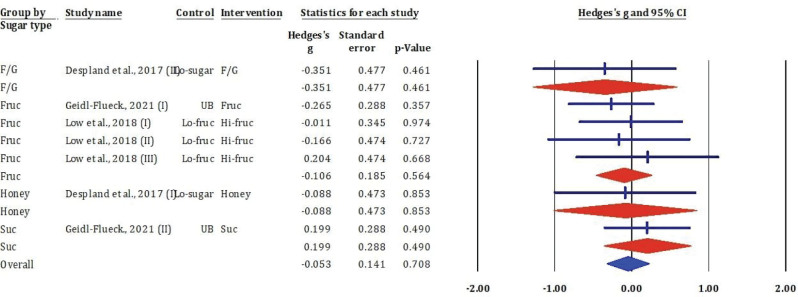
Effects of F/G, fructose, honey, and sucrose on VLDL-c: meta-analysis results (*n* = 7).

Figure 7 showed the results of the meta-analysis evaluated seven RCT comparisons examining the effect of added sugars on VLDL-c. The overall pooled estimate indicated no statistically significant change in VLDL-c following sugar intervention (Hedges’s *g* = –0.053, SE = 0.141, *P* = 0.708), with the 95% CI crossing zero.

Subgroup analyses showed consistently non-significant findings across all sugar types. Fructose-glucose mixtures, as reported by Despland et al. (25), resulted in identical moderate negative effect sizes (Hedges’s *g* = –0.351, *P* = 0.461), suggesting a potential but inconclusive reduction in VLDL-c. Fructose-only interventions produced a mix of weak positive and negative effects, with none reaching statistical significance (*P* > 0.3). Similarly, honey and sucrose interventions showed negligible impact on VLDL-c levels (Hedges’s *g* = –0.088 and 0.199, respectively; both *P* > 0.4).

Overall, these findings suggest that acute or short-term intake of fructose, sucrose, or their combinations does not significantly alter circulating VLDL-c levels. This contrasts with the more pronounced effects observed on LDL-c and TG, highlighting possible differential metabolic responses across lipid fractions.

### Effects of fructose-glucose mixtures, fructose, honey, and sucrose on serum triglycerides

**Fig. 8:**
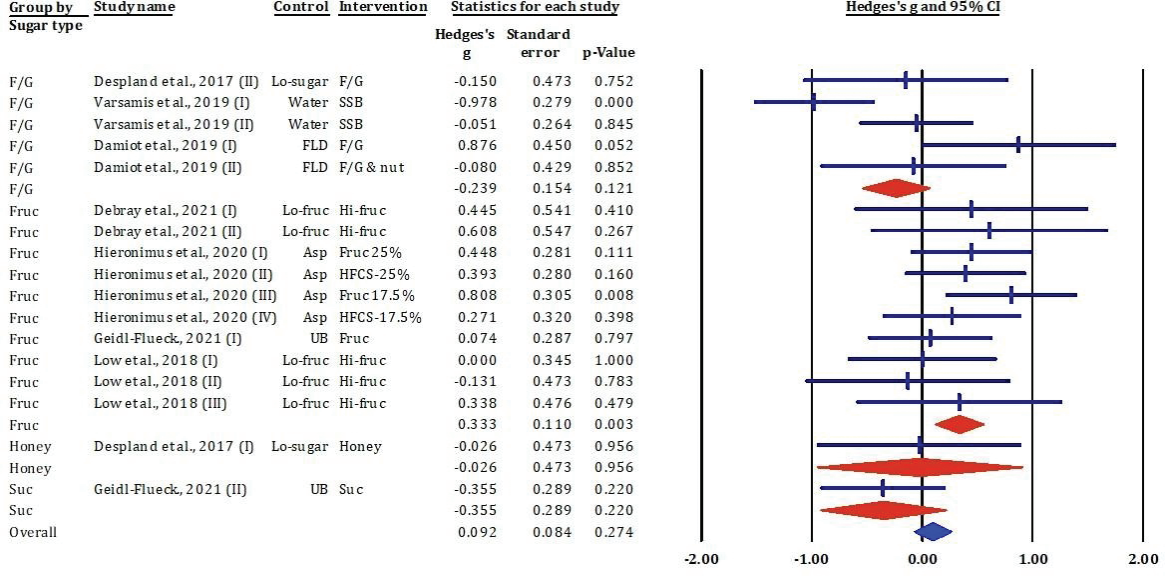
Effects of F/G, fructose, honey, and sucrose on serum TG: meta-analysis results (*n* = 17).

Figure 8 presents a forest plot summarising the standardized mean differences (Hedges’ g) and 95% CIs from individual studies investigating the effects of various sugar types – F/G, fructose, honey, and sucrose – on serum TG levels.

In the F/G group, most comparisons (e.g. 23, 25) showed small and non-significant effects on serum TG levels (Hedges’ *g* ranging from −0.28 to 0.15, *P* > 0.05), except for Varsamis et al. (22) (I), where a significant reduction was observed (Hedges’ *g* = −0.978, *P* < 0.001). In contrast, results within the fructose-only subgroup were more variable. Notably, Low et al. (24) (III) reported a significant increase in TG (Hedges’ *g* = 0.713, *P* = 0.003), while other studies showed negligible or non-significant effects.

The single study using honey (25) and one using sucrose (14) reported non-significant differences in serum TG (Hedges’ *g* = −0.365 and −0.356 respectively, both *P* > 0.05).

Overall, the meta-analysis indicated a small, non-significant pooled effect across all sugar types (Hedges’ *g* = 0.092, 95% CI: −0.072 to 0.255, *P* = 0.274), suggesting that consumption of these sugar types, in the contexts studied, did not significantly alter serum TG concentrations.

### Effects of fructose-glucose mixtures, fructose, honey, and sucrose on non-esterified fatty acids

**Fig. 9:**
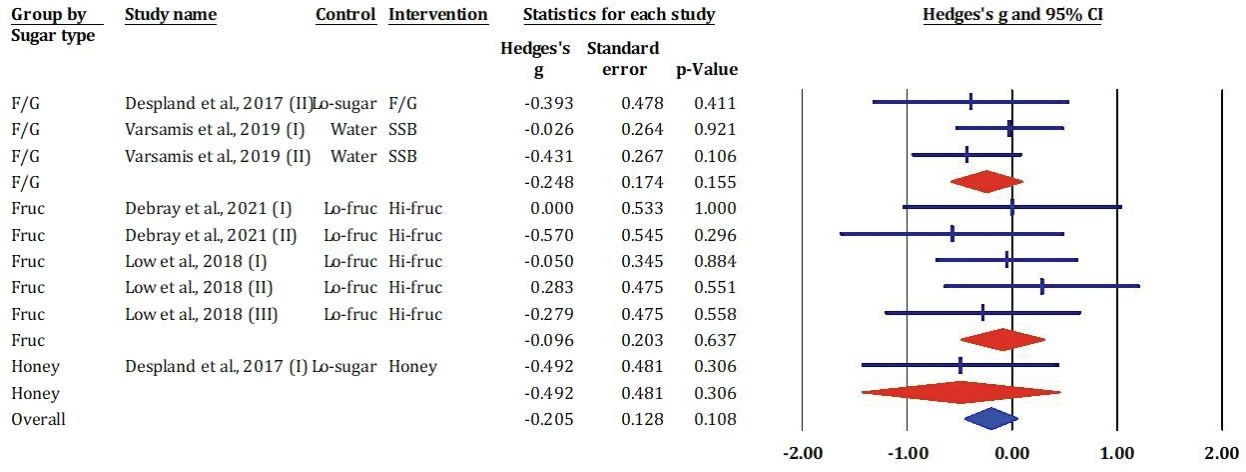
Effects of F/G, fructose, honey, and sucrose on NEFAs: meta-analysis results (*n* = 9).

Figure 9 displays a forest plot of the effects of different sugar types – F/G, fructose Fruc, and honey – on NEFAs concentrations, measured as standardised mean differences (Hedges’ *g*) with 95% CIs.

In the F/G subgroup, all three included comparisons (22, 25) showed small and non-significant effects on NEFA levels, with Hedges’ *g* ranging from −0.393 to −0.026 (*P* > 0.10). Similarly, studies in the fructose subgroup (20, 24) reported negligible and statistically non-significant changes in NEFAs.Notably, the largest effect was observed in a study conducted by Despland et al. (25) (Honey vs. Lo-sugar), with a moderate reduction in NEFAs (Hedges’ *g* = −0.492), though this was not statistically significant (*P* = 0.306).

The overall pooled estimate across all sugar types indicated a small, non-significant reduction in NEFA concentrations (Hedges’ *g* = −0.205, 95% CI: −0.455 to 0.045, *P* = 0.108). These findings suggest that short-term intake of these sugars does not significantly affect fasting or postprandial NEFA concentrations under the conditions studied.

### Effects of fructose-glucose mixtures, fructose, honey, and sucrose on uric acid

**Fig. 10:**
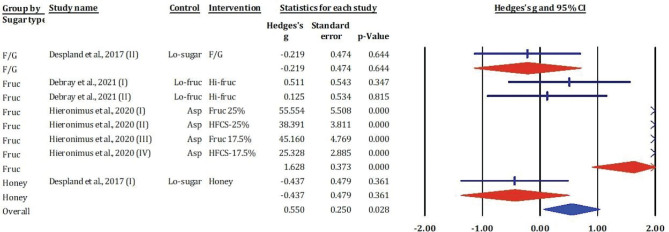
Effects of F/G, fructose, honey, and sucrose on uric acid: meta-analysis results (*n* = 8).

Figure 10 showed results from the meta-analysis included eight study comparisons assessing the impact of various dietary sugars on serum uric acid concentrations. The overall pooled effect size indicated a statistically significant increase in uric acid following sugar consumption (Hedges’s *g* = 0.550, SE = 0.250, *P* = 0.028), suggesting that added sugars – particularly fructose – may contribute to hyperuricemia.

Subgroup analyses revealed that the most pronounced effects were associated with fructose interventions. Several trials conducted by Hieronimus et al. (21) demonstrated remarkably high effect sizes with statistical significance (e.g. Hedges’s *g* = 55.554 for fructose 25%, *P* < 0.001; *g* = 38.391 for HFCS-25%, *P* < 0.001), although these unusually large values may reflect outlier data or measurement scale issues requiring further scrutiny. Other fructose interventions (20) reported moderate, though non-significant, increases in uric acid. In contrast, F/G and honey showed negligible or negative effects (e.g. *g* = –0.219 and –0.437, respectively; *P* > 0.3).

### Publication bias assessment

**Fig. 11:**
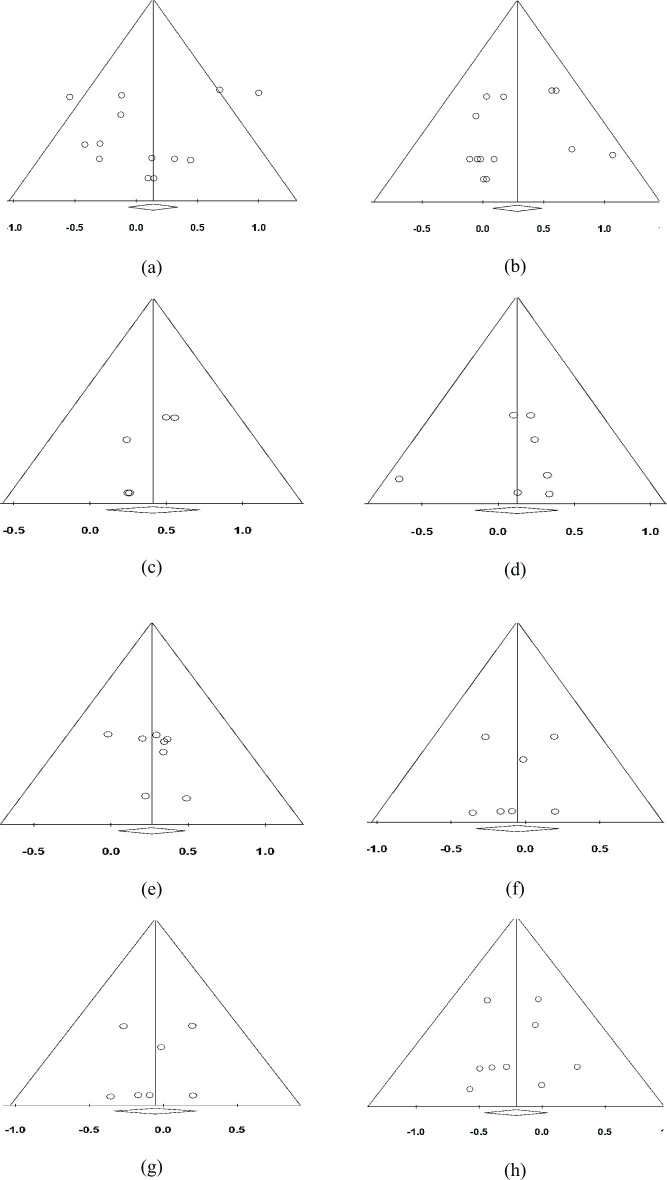
Funnel plot of publication bias for parameter (a) FBG, (b) Serum insulin, (c) TC, (d) HDL-c, (e) LDL-c, (f) VLDL-c, (g) Serum TG, and (h) NEFAs, where x-value is Hedge’s g and y-value is standard error.

Publication bias represents a significant concern in meta-analyses, potentially distorting results by favouring studies reporting positive outcomes. Assessment of publication bias was conducted to ensure the validity of the meta-analytic findings across key biomarkers, including blood glucose, serum insulin, and lipid fractions. (see Table 3 & Fig. 11). Egger’s regression test and Begg’s rank correlation test were employed to detect potential bias. For all biomarkers analysed, *p*-values exceeded 0.05, indicating no statistically significant evidence of publication bias. These results support the robustness of the meta-analysis and suggest that the overall findings are unlikely to be influenced by selective reporting.

**Table 3:** Publication bias assessment

Parameter	*P*-value using Begg’s test	*P*-value using Egger’s test	Fail-safe Number (Nfs)
Blood glucose	0.62550	0.40779	0
Serum insulin	0.46410	0.41441	8
Total cholesterol	0.62421	0.11215	3
HDL cholesterol	0.45269	0.68153	0
LDL cholesterol	0.13765	0.35568	5
VLDL cholesterol	0.45269	0.72859	0
Serum triglycerides	0.50984	0.40546	0
NEFA	0.29715	0.86096	0

While formal testing did not reveal evidence of publication bias (*P* > 0.05), the relatively low calculated fail-safe numbers suggest that the current findings might be sensitive to the inclusion of unpublished null studies. The fail-safe number represents the estimated number of such studies required to reduce the observed effect to non-significance. Therefore, although this meta-analysis provides robust and credible results based on the included data, future research incorporating a broader spectrum of studies is warranted to enhance the generalisability and confirm the observed biomarker relationships with greater certainty.

## Discussion

Dietary fructose is commonly consumed through fructose-containing caloric sweeteners (sucrose, HFCS) as well as from natural sources such as fruits and honey. These sources typically provide fructose and glucose in approximately equimolar proportions. The metabolic processing of fructose is influenced by several factors notably the concurrent intake of glucose or other nutrients, which can alter the secretion and activity of glucoregulatory hormones (26). Clinical trials in humans have associated fructose intakes exceeding 50 g per day – equivalent to approximately 8–12% of total energy intake – with an increased risk of metabolic syndrome (MetS) (27). In contrast, fructose derived from natural sources (fruits and vegetables) typically contribute only 5% of total energy intake or approximately 30 g per day (28). Growing global concerns has focused on ultra-processed or industrialised foods as primary contributors to the development of MetS, due to their widespread availability and affordability.

Our findings indicate that sucrose consumption is associated with significant increases in FBG and serum insulin concentrations, whereas fructose consumption alone did not elicit significant changes in these parameters. These results are inconsistent with prior research suggesting that free fructose may pose a greater risk for metabolic syndrome components than sucrose (29). Furthermore, this meta-analysis demonstrates that fructose, when consumed as fructose-sweetened beverages or HFCS, exerts a substantial effect on serum uric acid and triglyceride levels, exceeding that of other fructose-containing monosaccharides.

A key distinction between fructose and glucose metabolism lies in fructose’s ability to bypass phosphofructokinase, the primary rate-limiting enzyme of glycolysis. Consequently, fructose is preferentially utilised for glycerol and fatty acid synthesis via DNL, a process that occurs more rapidly than glucose conversion (30). Furthermore, fructose consumption can impair triglyceride clearance by lipoprotein lipase (LPL) (31). Diets high in fructose have been shown to elevate VLDL-associated plasma TG in humans (31–33), potentially leading to increased VLDL remnant concentrations and hepatic remnant uptake (34).

A positive correlation was observed between fructose consumption and serum triglyceride concentrations. In contrast, consumption of honey and sucrose did not significantly affect serum triglyceride concentrations. Previous studies have demonstrated that acute hepatic metabolism of fructose enhances substrate availability for DNL, leading to elevated plasma triglyceride levels following fructose ingestion (35).

Within 4 to 6 h of ingestion, a minor fraction of fructose-derived carbon is directly converted into lipids (36, 37). However, chronic fructose consumption has been shown to stimulate hepatic lipogenesis through the activation of specific metabolic pathways. Fructose metabolism in the liver leads to an accumulation of intrahepatic carbohydrate derived metabolites, which acts as nutritional signals regulating key transcription factors including carbohydrate response element-binding protein (ChREBP) and sterol regulatory element-binding protein 1c (SREBP1c). These transcription factors, along with associated coactivators, upregulate the expression of genes involved in lipogenesis and other metabolic processes (38).

Approximately 15% of intrahepatic lipids originate from dietary fat (39), with saturated fatty acids playing a significant role in hepatic lipid accumulation. Chylomicron triglycerides (TAG) are hydrolysed by LPL, facilitating the uptake of fatty acids for storage or oxidation in peripheral tissues. Fatty acid spillover from chylomicron-triglyceride lipolysis represents one pathway by which dietary lipids may accumulate in the liver (40). In this meta-analysis, fructose consumption was not associated with significant changes in circulating NEFAs levels. Under fasting conditions, NEFAs are released from adipose tissue triacylglycerol via LPL activity in capillaries, serving as a primary energy fuel. The activities of both LPL and hormone-sensitive lipase (HSL) are regulated by insulin and acylation-stimulating protein (ASP) (41).

Varsamis et al. (22) reported a 13% reduction in the total area under the curve (tAUC) for NEFA following consumption of SSBs compared to water. This reduction is likely attributable to the lipogenic effect of fructose and its influence on insulin secretion and activity (42). Conversely, Despland et al. (25) and Debray et al. (20) observed no significant changes in NEFAs levels following the consumption of honey or fructose. These inconsistencies in the literature may explain the non-significant overall effect observed in the present meta-analysis. Therefore, additional well-controlled studies are needed to clarify the specific impact of fructose consumption on NEFA concentrations.

This meta-analysis demonstrates that fructose consumption significantly increased uric acid concentrations. Subjects with hereditary fructose intolerance (HFI) exhibited elevated postprandial uric acid levels following a high-fructose diet (20). Similarly, a significant increase in 24-h uric acid was observed after a 14-day intervention with a high-fructose or HFCS diet, providing 25% of daily energy requirements (21). Conversely, Despland et al. (25) reported no significant effect of honey or F/G on fasting uric acid levels. However, long-term fructose consumption may elevate the risk of hyperuricemia and gout, as suggested by Jamnik et al. (43). The metabolism of fructose involves the reduction of adenosine triphosphate (ATP), resulting in the accumulation of inosine monophosphate (IMP). This process stimulates the activity of adenosine deaminase and xanthine oxidase, thereby enhancing uric acid production. Our meta-analysis revealed that consumption of fructose, sucrose, and F/G was associated with a significant increase in LDL-cholesterol levels, while no significant effect was observed on HDL-cholesterol levels. Previous studies have shown that short-term high fructose consumption, even over a period of 2 weeks, can elevate fasting LDL-c concentrations by approximately 5–6% (19). Similarly, Geidl-Flueck et al. (14) reported that daily consumption of 80 g/day of either fructose or sucrose over 7 weeks significantly enhanced hepatic lipogenic activity.

The effect of fructose on LDL-c is primarily mediated through its rapid hepatic metabolism to glyceraldehyde-3-phosphate (GAP) and dihydroxyacetone phosphate (DHAP), which are subsequently converted to acetyl-CoA and undergo DNL pathway. In addition to serving as metabolic substrates, fructose stimulates lipogenesis through the activation of transcription factors, including ChREBP and SREBP1c. The activation of these lipogenesis transcription factors results in increased VLDL production and secretion, contributing to elevated circulating LDL-c levels (9).

## Conclusions

This meta-analysis evaluated the metabolic effects of various dietary sugars – including fructose, sucrose, and F/G mixtures – on glycaemic control, lipid profiles, NEFAs, and serum uric acid. The findings demonstrate that while added sugars have minimal impact on FBG, insulin, TG, NEFA, HDL-c, and VLDL-c under acute or short-term conditions, they are associated with significant increases in TC and LDL-c – key indicators of atherogenic risk. Notably, fructose and sucrose consistently contributed to elevated TC and LDL-c concentrations. Furthermore, select fructose interventions, particularly those involving HFCS, were linked to pronounced increases in uric acid, though extreme effect sizes warrant cautious interpretation due to potential outliers or methodological inconsistencies.

Taken together, the evidence suggests that while short-term sugar intake may not acutely disrupt glucose-insulin homeostasis or NEFAs levels, habitual consumption of added sugars – especially fructose and sucrose – may adversely influence lipid metabolism and uric acid concentrations, thereby increasing long-term cardiometabolic risk. Future studies should focus on chronic intake, dose-response relationships and population-specific vulnerabilities to refine dietary recommendations.
